# Galectins in Endothelial Cell Biology and Angiogenesis: The Basics

**DOI:** 10.3390/biom11091386

**Published:** 2021-09-20

**Authors:** Victor L. Thijssen

**Affiliations:** Cancer Center Amsterdam, Department of Radiation Oncology, Amsterdam UMC Location VUmc, De Boelelaan 1118, 1081 HV Amsterdam, The Netherlands; v.thijssen@amsterdamumc.nl

**Keywords:** vasculature, gene expression, tube formation, sprouting, VEGF, integrins, galectin, extracellular matrix, microenvironment

## Abstract

Angiogenesis, the growth of new blood vessels out of existing vessels, is a complex and tightly regulated process. It is executed by the cells that cover the inner surface of the vasculature, i.e., the endothelial cells. During angiogenesis, these cells adopt different phenotypes, which allows them to proliferate and migrate, and to form tube-like structures that eventually result in the generation of a functional neovasculature. Multiple internal and external cues control these processes and the galectin protein family was found to be indispensable for proper execution of angiogenesis. Over the last three decades, several members of this glycan-binding protein family have been linked to endothelial cell functioning and to different steps of the angiogenesis cascade. This review provides a basic overview of our current knowledge regarding galectins in angiogenesis. It covers the main findings with regard to the endothelial expression of galectins and highlights their role in endothelial cell function and biology.

## 1. Introduction

With an estimated length of at least 100,000 kilometers (±60,000 miles), the adult human vasculature forms an immense infrastructure encompassing all blood vessels, ranging from the large arteries and veins to the countless number of small capillaries. This vast vascular bed ensures that all organs, tissues, and cells in the body have access to sufficient amounts of oxygen and nutrients and that waste materials can be disposed of. In addition, platelets and blood-borne cells, such as leukocytes, are able to travel to all parts of the body via the vasculature. The key players that are involved in building, maintaining, and providing functionality to the blood vessel system are the endothelial cells. These cells are of mesodermal origin and they cover the inner surface of all blood vessels. As such, the vascular endothelium serves as the main interface between all components in the blood and the underlying tissues. Consequently, endothelial cells participate in several biological processes, e.g., coagulation, inflammation, and transendothelial transport/migration [1]. Moreover, in case of a demand for new vessels, the endothelial cells can be triggered to start the formation of new blood vessels, a process referred to as angiogenesis. Angiogenesis is not only an intricate part of different physiological processes, e.g., the menstrual cycle, embryogenesis, or wound healing, it is also involved in different pathologies, including cancer [2]. In fact, 50 years ago it was shown that tumor tissues, once they reached a few cubic millimeters, depended on activation of angiogenesis in order to maintain growth [3,4]. If tumor cells fail to induce angiogenesis, the growing tumor mass is provided with insufficient oxygen and nutrients and remains dormant [5]. Consequently, activation of tumor angiogenesis is considered a hallmark of cancer and targeting this process has been recognized as a potent strategy for cancer therapy [6,7].

Angiogenesis, both in the physiological and pathological context, is a complex and multistep process during which endothelial cells respond to a multitude of external and internal signals [7]. These signals trigger endothelial cells to adopt different phenotypes that ultimately result in the formation of new blood vessels. It is now well recognized that several galectins are involved in facilitating different endothelial activities during angiogenesis [8,9,10]. The current review will provide a basic overview of the current knowledge regarding the role of galectins in endothelial cell biology and angiogenesis.

## 2. Endothelial Galectin Expression

**Figure 1:**
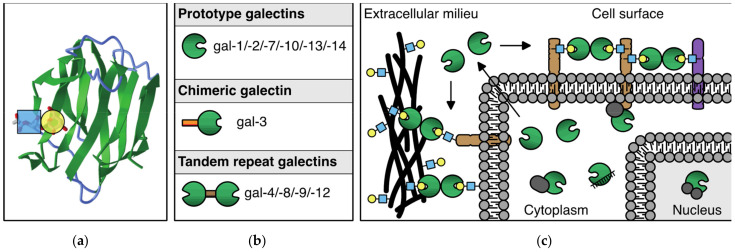
The galectin protein family. (**a**) Cartoon of the anti-parallel beta-sheet structure forming the carbohydrate recognition domain of galectin-1. On the left, the interaction of a LacNAc(N-acetyllactosamine) moiety in the binding groove is shown. (**b**) Overview of the 11 mammalian galectins that are expressed in humans. See text for explanation of the subgroups. (**c**) Schematic representation of the (extra)cellular location of galectins. In the extracellular environment and on the cell surface, galectins can interact with glycoconjugates to facilitate, e.g., cell–ECM and cell–cell interactions. In addition, galectins can mediate interactions between molecules in the cell membrane. In the cytosol and nucleus, galectins can engage in (mostly) glycan-independent protein/protein interactions involved in, e.g., signaling and mRNA splicing.

The mammalian galectin family comprises 15 members, 11 of which were also found expressed at the protein level in humans. These glycan-binding proteins share a so-called carbohydrate recognition domain (CRD) of approximately 130 amino acids, which is composed of two antiparallel beta-sheets that fold in a beta-sandwich. The beta-sandwich structure is slightly curved forming a groove in which carbohydrate binding occurs. Main interactions with beta-galactoside containing glycans involve a core-binding site inside the groove that contains several evolutionary conserved amino acids (Figure 1a). Glycan-binding specificity and affinity of each galectin are further mediated through small structural differences within (and outside) the binding groove. Based on the number and structural arrangement of the CRDs, galectins can be classified into three subgroups (Figure 1b), i.e., prototype galectins (single CRD; gal-1/-2/-7/-10/-13/-14), chimeric galectins (single CRD with an N-terminal non-lectin domain; gal-3), and tandem repeat galectins (two linked CRDs; gal-4/-8/-9/-12). Their ability to engage in glycan-dependent, as well as glycan-independent interactions, allows them to exert multiple functions in many different biological processes. Indeed, galectins are expressed by many different cell types where they can be found intracellularly and/or extracellularly (Figure 1c). For an extensive and more in-depth background on the structure, glycan-binding, and function of galectins, see [11,12,13]).

**Figure 2:**
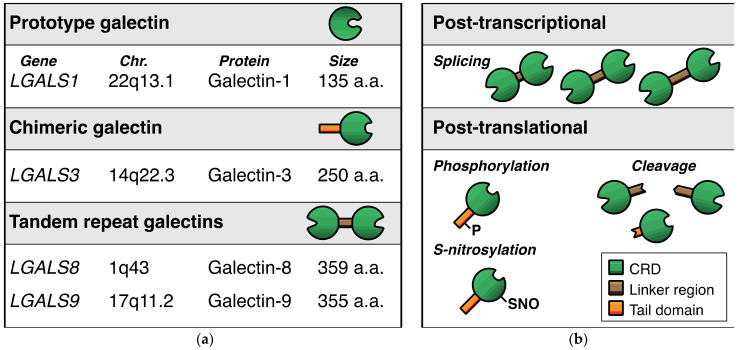
Endothelial galectins. (**a**) Schematic representation of the four dominant galectins that are expressed by endothelial cells. (**b**) Overview of the main post-transcriptional and post-translational modifications that occur in endothelial galectins. Note that the modifications shown here are illustrative and do not represent the actual location of modification in the respective proteins.

With regard to the expression of galectins in endothelial cells, we performed a broad galectin-profiling study in 2008 showing that the endothelial expression of galectins is mainly restricted to galectin-1, -3, -8, and -9 (Figure 2a) [14]. The mRNA expression levels of three other galectins (galectin-2, -4, -12) were low and not confirmed at the protein level, while mRNA expression of the remaining galectins (galectin-7, -10, -13, -14) could not be detected at all. The findings of this extensive galectin profiling study corroborated previous observations [15,16,17,18] and were later confirmed by different research groups in endothelial cells from variable origins [19,20,21,22,23,24]. Only recently, it was reported that galectin-2 protein expression was also detectable in endothelial cells, albeit in a specific context, i.e., in fetal endothelial cells in the placenta of women with gestational diabetes mellitus [25]. While this needs further confirmation, it indicates that the cellular context can control specific endothelial galectin expression. In line with this, galectin-8 expression appears to be higher in primary isolated lymphatic endothelial cells when compared to regular endothelial cells [19,26]. Likewise, galectin-3 expression was reported to be higher in endothelial progenitor cells as compared to normal endothelial cells [27]. Furthermore, we (and others) have shown that several cytokines, growth factors, and other molecules can alter galectin expression levels in endothelial cells (Table 1) [9]. For example, galectin-8 and galectin-9 expressions are reduced in serum-activated primary isolated endothelial cells as compared to non-activated counterparts [22]. At the same time, treatment with interferon gamma (IFNγ) can trigger the endothelial expression of galectin-9 [23,24,28,29]. IFNγ, as well as other cytokines, was also shown to increase endothelial galectin-1 expression [16,30], and more recently, cathepsin L was found to induce endothelial expression of galectin-1 [31]. The expression of galectin-3 by endothelial cells was shown to be induced by, e.g., matrix component fibronectin [32], advanced glycosylation end products [33], and interacting neutrophils [34]. It is important to note that the overview presented in Table 1 is likely far from complete, as it is still poorly understood which and how environmental triggers affect endothelial galectin expression. This is illustrated by the observation that many cancer tissues—often characterized by an aberrant microenvironment—display altered expression of endothelial galectins (for overview see [9]). For example, we have shown that galectin-9 expression, which is reduced in activated endothelial cells [14], is significantly increased in the tumor endothelium of different cancer types [35]. Since tumor cells secrete many different factors to modulate their microenvironment, including the immune infiltrate and the vasculature, it can be anticipated that the list of proteins provided here merely represents the tip of the iceberg when it comes to regulation of endothelial galectin expression. 

To further complicate matters, it has been shown that extracellular triggers, such as cytokines, growth factors, or hypoxia, can alter the glycosylation patterns on the endothelial cell surface, which in turn affects the binding of galectins to the cells [36,37]. For example, hypoxia was shown to increase the presence of β1-6GlcNAc-branched N-glycans, poly-LacNAc structures, and fucosylated glycans on the endothelial cell surface, while α2-6 sialylation and α2,3-sialylated moieties are reduced [36,38]. Such alterations change the permissiveness of the endothelial cell towards specific galectin binding, which in turn affects how galectins control endothelial cell functionality.

At the same time, galectins can trigger the endothelial expression and release of cytokines and growth factors [21,39,40,41]. All of this points towards a complex relationship between the microenvironment and endothelial galectin expression. It is an ongoing challenge to unravel this relationship, in particular in the in vivo context where multiple triggers can simultaneously influence the endothelial cell phenotype.

It is also important to realize that endothelial galectin expression is not only controlled at the transcriptional level, but also at the post-transcriptional and post-translational level (Figure 2b). For example, alternative splicing has been shown to occur for tandem repeat galectin-8 and galectin-9. In endothelial cells, the alternative splicing can give rise to up to 3 different protein isoforms by affecting the length of the linker region between the two CRDs [14,20,22,29,42,43]. While the regulatory mechanisms that control the alternative splicing remain elusive, we observed that cytokines and growth factors could affect the mRNA expression levels of the different endothelial galectin-9 splice variants in vitro [22]. Whether it is also true for endothelial galectin-8, and how this is regulated in endothelial cells in vivo, requires further investigation. 

The need for more research also applies to the role of post-translational modifications of galectins in endothelial cells. Different protein modifications with different functional effects on galectins have been reported, including proteolytic cleavage, phosphorylation, and S-nitrosylation [44,45,46,47,48,49,50,51]. With regard to angiogenesis, it was shown that proteolytic processing of galectin-3 influences the angioregulatory activity of the protein (see also next section) [52]. We also found that the two distinct galectin-9 CRDs, which can be generated upon proteolytic cleavage [51], have different effects on endothelial cell function [23]. However, a comprehensive insight in the post-translational modifications of endothelial galectins, how it is regulated, and how it affects endothelial cell function is still lacking.

Finally, an important aspect that should be taken in consideration when studying endothelial galectin expression is the cellular localization. As briefly mentioned before, galectins can be located intracellularly and extracellularly. In fact, galectins can be found in specific compartments of a cell, including the nucleus, the cytoplasm, and the cell membrane [14,20,53,54]. In addition, galectins can be secreted into the extracellular milieu [21,39,41]. With regard to the cellular localization and secretion of galectins, again, environmental clues that regulate endothelial activity appear to play an important role. For example, activation of cultured endothelial cells by high serum conditions or by a tumor conditioned medium was shown to increase cell surface exposure of galectin-1, -8, and -9 [14,30]. In line with this, the surface translocation of endothelial galectin-9 can be triggered by IFNγ [28,29] while secretion of galectin-8 has been linked to treatment with LPS [21]. Furthermore, endothelial cells in tumor tissues show altered cellular location of galectins as compared to normal endothelium [14,20]. All of this further supports the concept that the microenvironment is a key regulator of endothelial galectin expression as it provides most of the signals to which the endothelial cells respond.

## 3. Galectins in Endothelial Cell Function and Angiogenesis

As evident from the previous section, the expression of endothelial galectins is controlled by many different factors. Such a complex level of regulation suggests that galectins are involved in different aspects of endothelial cell function. Indeed, research over the last three decades has shown that adequate expression and function of galectins is required in multiple endothelial cell activities related to, e.g., inflammation and immunomodulation [55], coagulation [56], and angiogenesis [9,57,58,59]. While there is increasing interest in the immunomodulatory functions of endothelial galectins [24,60], this review will solely focus on the angioregulatory role. In particular, the role of the individual endothelial galectins in cellular functions related to angiogenesis will be highlighted.

### 3.1. Galectin-1

The first studies reporting on endothelial galectin-1 expression appeared around the 1990s in the previous century [15,17,61,62]. While these findings hinted towards a possible function in angiogenesis, the first clear evidence that directly linked galectin-1 to endothelial cell biology was provided by us in 2006. Using a protein–protein interaction screen, we identified galectin-1 as the target protein of a synthetic peptide inhibitor of angiogenesis [63]. Subsequent research showed that galectin-1 was essential for different endothelial cell functions during angiogenesis, in particular cell proliferation and migration [63]. Nowadays, it is well established that endothelial cells in vitro as well as in vivo angiogenesis rely on galectin-1 [31,64,65,66,67,68,69,70]. Only recently, the importance of galectin-1 in endothelial cell biology was again confirmed in a study that explored vascular remodeling after cerebral ischemia [71]. This study also reiterated the important link between galectin-1 and the angiostimulatory protein vascular endothelial growth factor (VEGF) by showing an association between galectin-1 and VEGF/VEGF receptor expression in endothelial cells [71]. Previous work had already shown that galectin-1 can delay endocytosis of VEGFR2 [72] and that binding of galectin-1 to the VEGFR2 co-receptor neuropilin-1 enhanced receptor phosphorylation and downstream signaling [64]. Moreover, Croci et al. found that altered glycosylation of VEGFR2 allows galectin-1 to activate receptor signaling in VEGF refractory tumors [36]. Importantly, this was linked to interactions of galectin-1 with non-sialylated N-linked glycans on the VEGF receptor [36], indicative of an important role of endothelial cell glycosylation in the sensitivity to galectins. Interestingly, it was also recently suggested that galectin-1 might interact with VEGF mRNA transcripts, which might interfere with VEGF translation and/or secretion [73]. This could be related to the possible role of galectin-1 in splicing [74] but needs further validation. Nevertheless, it is evident that galectin-1 can induce endothelial cell activation and control or even replace the angiostimulatory activity of VEGF. Altogether, these findings show that the galectin-1/VEGF/VEGFR2 axis represents an important route for inducing and maintaining endothelial cell activation. Of note, galectin-1 was also shown to regulate vascular permeability involving neuropilin-1/VEGFR1 mediated signaling [75].

Apart from the interactions with VEGF (co)receptors, galectin-1 was also shown to bind to CD146 (melanoma cell adhesion molecule; MCAM), which resides on the endothelial cell surface. It was suggested that this interaction prevented galectin-1-induced apoptosis with CD146 serving as a galectin-1 scavenger molecule [76]. While the observation that galectin-1 can induce endothelial cell apoptosis appears contradictory to its angiostimulatory role, it is important to realize that high concentrations of galectin-1 were used in this particular study (millimolar range). Indeed, the activity of galectin-1, in particular in relation to glycan-binding functionality, is dependent on the ability to form homodimers [77,78,79,80]. At too low concentrations, insufficient numbers of dimers will be formed, while at too high concentrations, an excess of dimers might interfere with effective crosslinking of glycoproteins. Therefore, the effects of galectin-1 on endothelial cells (and on other cells) were found to be concentration-dependent with inhibitory effects at high concentrations [65,81,82]. This biphasic activity should always be taken into account when studying the function of galectin-1 in endothelial cell biology and angiogenesis.

Finally, many other proteins were identified that engage in either protein-carbohydrate or protein-protein interactions with galectin-1 [83]. While some of these proteins have known functions in endothelial biology, e.g., thrombospondin, integrins α1β1 and α5β1, it is still poorly understood whether and how these interactions contribute to endothelial cell function or angiogenesis.

### 3.2. Galectin-3

The first compelling evidence that galectin-3 is involved in angiogenesis was provided by Nangia-Makker et al. The authors observed increased endothelial cell tube formation in the presence of galectin-3 as well as a higher number of blood vessels in Matrigel plugs in mice that contained galectin-3 compared to Matrigel alone [84,85]. The angiostimulatory activity, including enhanced migration, proliferation, and in vivo angiogenesis, was later confirmed by others [72,86,87,88,89]. However, the context is important, as galectin-3 was also shown to increase endothelial cell dysfunction in the presence of oxidized low-density lipoprotein [90] and to inhibit endothelial cell proliferation [91].

Similar to galectin-1, galectin-3 can delay VEGFR2 endocytosis and stimulate VEGFR2 phosphorylation and signaling [72,86,87]. In fact, when applied together, galectin-1 and galectin-3 were also shown to trigger VEGFR1 signaling in endothelial cells [72]. Next to increased VEGFR2 signaling, the angiostimulatory activity of galectin-3 was recently also linked to the protein’s ability to interact with JAG1, a NOTCH1 ligand. Dos Santos et al. described that binding of galectin-3 to JAG1 increased the half-life of the latter resulting in enhanced JAG1/NOTCH1 signaling and stimulation of sprouting angiogenesis [92]. Interestingly, a galectin-3/JAG1/NOTCH1 signaling axis has also been linked to transdifferentiation of pulmonary artery-derived endothelial cells into a smooth muscle cell-like phenotype [91]. In line with this, it has recently been described that galectin-3 can regulate endothelial-to-mesenchymal transition of human lung micro-endothelial cells [93]. Although it requires further investigations, it is tempting to speculate that galectin-3 plays a role in regulating the balance between a migratory and proliferative phenotype of endothelial cells, which is key during angiogenesis.

Of note, it was reported that the angiogenic activity of galectin-3 is lost upon removal of the N-terminal tail [87,94]. In line with this, it was shown that proteolytic removal of a large part of the tail by PSA hampers the angiogenic activity of galectin-3 [48]. At the same time, proteolytic cleavage by MMP within the N-terminal tail of galectin-3 can stimulate the angiogenic activity [52]. Moreover, aminopeptidase N (CD13) was suggested to enhance the angiostimulatory activity of galectin-3 by proteolytic processing [95]. Apparently, galectin-3, and in particular the non-CRD tail, is susceptible to proteolytic cleavage, which is important for the activity of the protein during angiogenesis. Whether galectin-3 processing affects both VEGFR2- and JAG1/NOTCH1-mediated signaling requires further investigation. It is however tempting to speculate that modifications of the tail region control the ability of galectin-3 to oligomerize, which might differentially affect both signaling pathways.

The ability of galectin-3 to stimulate endothelial migration and tube formation has been linked to integrin-αV/β3. Galectin-3 was found to induce glycosylation-dependent clustering of integrin-αV/β3, resulting in enhanced FAK signaling [86]. Consequently, blocking integrin-αV/β3 hampered the VEGF-induced migration and tube formation by galectin-3 [86]. Galectin-3 was also found to form a complex with integrin-α3/β1 and the proteoglycan NG2, which could be involved in mediating the angiostimulatory activity of the latter [96]. More recently, a study by Sedláø et al. also suggested a role for glycan-independent effects of galectin-3/integrin interactions. The authors described that blocking antibodies targeting integrin-αV/β3, integrin-α5/β1, or integrin-α2/β1 could hamper endothelial cell adhesion to a galectin-3-coated surface [97]. All of these findings suggest an important link between galectin-3 and integrins in controlling endothelial cell biology, in particular with regard to endothelial cell migration and adhesion.

Apart from integrins, galectin-3 was shown to interact with other proteins on the endothelial cell surface, including CD31 (PECAM-1), CD146 (MCAM), CD144 (VE-cadherin), CD106 (endoglin) [41,98]. As described above, CD146 could serve as a galectin-1 scavenger molecule to hamper galectin-1-induced apoptosis [76]. The interaction of galectin-3 with CD146 was shown to activate AKT signaling and stimulate the release of cytokines by endothelial cells [41]. In addition, the CD146 interaction reduced endothelial cell migration [99]. Whether CD146 also serves as a galectin-3 scavenger or whether galectin-1/CD146 interactions affect cytokine release and migration remains to be studied. In addition, the functional consequences of the other galectin-3/protein interactions in endothelial cell biology are still largely unknown and should be further explored. 

### 3.3. Galectin-8

In contrast to galectin-1 and galectin-3, research on the role of galectin-8 in endothelial cell biology and angiogenesis is relatively sparse. Nevertheless, it was shown that this tandem repeat galectin is also involved in regulation of endothelial cell function (for excellent review see [57]). In part, the regulatory activity appears to be dependent on the endothelial cell phenotype and the presence of galectin-8-binding proteins that are associated with that phenotype. For example, in lymph endothelial cells, which display high expression of galectin-8, a glycosylated transmembrane protein called podoplanin was identified as an important binding partner. Podoplanin is a specific marker of lymphatic vessels and indeed, lymph endothelial cells were able to bind to surface-immobilized galectin-8 while regular endothelial cells were not [19]. At the same time, in a tube formation assay on collagen, galectin-8 was inhibitory towards lymph endothelial cells [19] while stimulatory towards regular endothelial cells [20]. In the latter, CD166 (ALCAM) was identified as a binding partner suggesting that the interaction of galectin-8 with specific endothelial cell surface molecules determines the angioregulatory function of the protein. In line with this, Hadari et al. showed that endothelial cell binding to vitronectin was hardly enhanced by galectin-8 since this adhesion is mediated through integrinαVβ3, which only shows limited interaction with galectin-8 [100]. Instead, galectin-8 was shown to interact with other integrin subunits present in endothelial cells, including α3, α5, and β1 [100,101]. As such, galectin-8 can be expected to differentially mediate cell adhesion and migration, depending on the presence of specific extracellular matrix components as well as certain endothelial cell surface molecules. The complexity of such interactions was shown by Chen et al., again, in the context of lymphangiogenesis. The authors not only confirmed the interaction between podoplanin and galectin-8, but also presented elegant data that supported a model in which galectin-8 clustered podoplanin with integrins α1β1/α5β1 in order to activate signaling pathways in lymphangiogenesis. By additional clustering of this complex with VEGR3, signaling was further potentiated [26]. Interestingly, galectin-8 was also shown to interact with CD44 [102], a surface molecule that is associated with angiogenesis [103,104] and that also binds podoplanin to promote tumor cell migration [105]. To what extent potential protein clusters of galectin-8/podoplanin/CDD44/integrins contribute to, e.g., endothelial cell migration, is currently not known.

Apart from a role in (lymph)endothelial cell adhesion and migration, galectin-8 has also been shown to induce a pro-inflammatory phenotype in endothelial cells, which was characterized by increased secretion of proinflammatory cytokines and increased binding of platelets [21]. More recently, it was suggested that galectin-8 enhanced the stimulatory effects of VEGF on endothelial cell proliferation and migration, but these effects were small. Moreover, the effects were only observed at the lowest concentration and galectin-8 alone did not stimulate proliferation and migration [106]. The clearest stimulatory effect of galectin-8 on angiogenesis, both with or without VEGF, was observed in the in vivo chorioallantoic membrane assay [106]. Whether these findings hint towards a function of galectin-8 in VEGF-signaling, similar as galectin-1 and galectin-3 needs further confirmation. In that regard, it has been shown that galectin-8 can bind to VEGFR2 [101].

Finally, galectin-8 was also shown to increase vascular permeabilization, similar as reported for galectin-1 [75]. However, the galectin-8 induced permeabilization appears to be triggered by a different pathway, i.e., activation of eNOS and disruption of adherens junctions through S-nitrosylation of p120 (Catenin Delta-1). Moreover, here, β1 integrins appeared to be involved [101].

### 3.4. Galectin-9

With regard to the regulation of endothelial cell function, galectin-9 is relatively a new kid on the block. Indeed, because endothelial galectin-9 expression was identified as an eosinophil chemoattractant [107] and was found induced by, e.g., IFNγ and viral RNA [29,108,109], the protein has been mainly studied in the context of immunomodulation [110,111]. With regard to angiogenesis, we have shown that exogenous application of galectin-9M, the dominant isoform in endothelial cells, hampers in vivo angiogenesis in the chicken chorioallantoic membrane assay [22,23]. In contrast, O’Brien et al. reported increased in vivo angiogenesis using a Matrigel plug assay in mice [112]. Although these appear as opposite findings, the Matrigel plug experiments actually confirmed the findings that galectin-9M serves as a chemoattractant for endothelial cells [22,112], while the CAM experiments confirmed the inhibitory effects of galectin-9M on proliferation and migration [22,23]. However, it should be noted that the effects of galectin-9M are concentration dependent and often show a biphasic effect, similar as described for galectin-1. In addition, the activity depends on the cellular activation status [23] as well as on the origin of the cells with primary endothelial cells being more sensitive (low nM range) compared to immortalized endothelial cells (high nM range) [22]. While this already indicates a complex regulatory role of galectin-9M in angiogenesis, matters are further complicated by the fact that multiple galectin-9 isoform exist and that the protein is subject to proteolytic cleavage as described previously. Indeed, when exploring the effects of the separate galectin-9 CRDs, we observed neutralization or even reversal of activity compared to galectin-9M. These effects again depended on endothelial cell activation status [23]. Thus, galectin-9 clearly regulates multiple aspects of endothelial cell biology and angiogenesis, but regulation is complex and the ultimate outcome depends on many intrinsic and extrinsic factors.

## 4. Summary and Outstanding Questions

Over the last thirty years, it has become evident that multiple galectin family members are expressed by endothelial cells and that these multifunctional proteins play key roles in endothelial cell biology and angiogenesis. As described here, endothelial galectin expression appears to involve primarily galectin-1, -3, -8, and -9. Importantly, different environmental conditions and triggers were found to affect the following: (i) the galectin expression level; (ii) the presence of (processed) galectin isoforms; and (iii) the cellular localization of galectins. Deciphering the mechanisms and pathways that control these aspects of endothelial galectin expression represents an important challenge for future research. This is particularly relevant in the context of disease as alterations in (vascular) galectin expression have been associated with different pathologies, including cancer [9,35].

**Figure 3:**
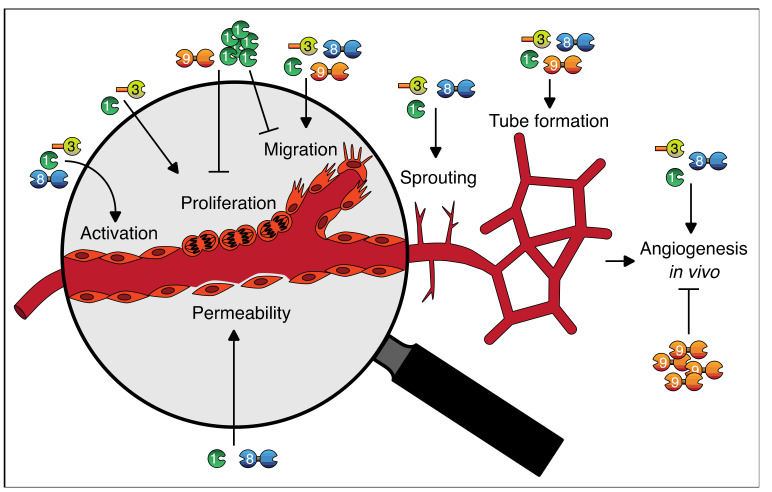
Graphical abstract of the roles of galectins in endothelial cell function and angiogenesis. See text and Table 2 for further explanation.

With regard to galectins as regulators of angiogenesis, many insights have been gained. It has become clear that galectins can regulate vessel permeability and vessel growth and that they contribute to multiple endothelial cell functions, including activation, proliferation, migration, tube formation, and sprouting (summarized in Table 2 and Figure 3).

In general, most galectins appear to be stimulatory but it is also evident that the effects are dependent on many different aspects, including the source and environmental context of the endothelial cell, the activation status, and importantly, the local concentration of the galectins. In that regard, it is important to realize that the findings described in this review were obtained using endothelial cells from a multitude of origins. This includes primary cells obtained from different tissues, e.g., umbilical, dermal, omental, pulmonary, aortic, as well as different endothelial cell lines from human or mouse origin. Moreover, different culture conditions were used with regard to, e.g., serum conditions, growth factors, matrix proteins. Since all of these differences can affect galectin expression and functionality, it is important to emphasize that the generalizations described here might be different for specific endothelial cells under specific conditions. In fact, deciphering the interplay between these aspects remains a future challenge since much of our current knowledge relies on in vitro findings or on studies using a single galectin. As it was shown that combined application of galectins can enhance the angiostimulatory activity [72], and it was suggested that galectins might engage in heterodimer formation [113], it is relevant to further explore the role of multiple galectins simultaneously in appropriate in vitro and in vivo models. In addition, while this review focused on the main endothelial galectins, recent findings have shown that other galectins can also play a role in regulating angiogenesis. For example, it was shown that galectin-12 expression is increased in adipose tissue under hypoxic conditions. Interestingly, hypoxia was also found to change the glycan-repertoire of endothelial cells, making them more permissive towards galectin-12 binding [38]. Subsequently, the authors showed that galectin-12 could act as a chemoattractant for endothelial cells and that the proteins stimulated tube formation in vitro. In addition, galectin-12 was required for adequate vascularization of adipose tissue in vivo [38]. In addition, galectin-13 has been linked to vascular remodeling, specifically of the uterine vasculature [114]. Although the latter awaits confirmation in humans, all these findings highlight the need for further research into the angioregulatory role of different galectins and specifically the relation with altered endothelial cell glycosylation patterns. As already recognized by Croci et al., a key future challenge will be to obtain a comprehensive insight in the endothelial cell glycome under physiological and pathophysiological conditions vessels, both in the preclinical and clinical settings. This will help to understand how galectins (as well as other glycan-binding proteins) are able to regulate vascular signaling programs and how to interfere with such programs in the context of therapy [37].

It is important to recognize that apart from the direct effects on angiogenesis, galectins can also trigger blood vessel growth indirectly. For example, galectins that are secreted or presented on the surface of endothelial cells can serve as chemoattractants for immune cells or as platelet activators [115]. This can trigger the release of angioregulatory molecules, such as cytokines and growth factors, which influence endothelial cell function and activity [115]. In addition, galectins in the extracellular milieu can serve as scavenger molecules for cytokines [116,117]. All of this further contributes to the angiomodulatory role of galectins. In particular, the width of galectin-cytokine interactions requires further research, as both protein families can exert angioregulatory as well as immunomodulatory functions [118,119,120]. As such, endothelial galectins hold a key position in the interface between the vasculature and immune cells, which should be further explored.

## 5. Future Perspectives

As described above, the research community has made considerable steps forward in understanding how galectins contribute to endothelial cell biology and angiogenesis. Nevertheless, many questions remain unanswered and there are sufficient challenges and questions that should be addressed in order to fully grasp the complex functions of galectins in vascular biology. At the same time, increasing insights in the function of galectins in the vasculature also provides opportunities, especially in the context of pathologies or diseases that are associated with aberrant vascular functionality, e.g., cardiovascular disease and cancer. Indeed, many galectin-targeting agents were developed by us and others, ranging from peptides, small molecules, and glycan-based ligands, to blocking antibodies, and have been shown to interfere with galectin functions during angiogenesis [63,66,70,84,121,122,123,124]. While a major future challenge is to translate these preclinical findings to clinical applications, it can be anticipated that such galectin-targeting molecules can be used for direct therapeutic applications as well as for indirect applications, including drug delivery and diagnostic imaging. Ultimately, this could help to develop novel and better treatment modalities for patients suffering from diseases that are associated with deregulated vascular galectin expression and or galectin dysfunction.

## Figures and Tables

**Table 1:** Regulators of endothelial galectin expression.

Galectin	Expression Induced by	Expression Reduced by
Galectin-1	IL-1β, IFNγ, TNFα, LDL, LPS, Cathepsin L, High serum ^b^	-
Galectin-3	IL-1β, fibronectin, AGEs, asialofetuin, neutrophil adhesion/transmigration	-
Galectin-8 ^a^	-	High serum ^b^
Galectin-9	IFNγ, IFNβ, IL-10, viral RNA	VEGF, IL-1, High serum ^b,c^

IL = Interleukin, IFN = Interferon, LDL = low-density lipoprotein, LPS = lipopolysaccharide, AGE = Advanced glycosylation end products, VEGF = vascular endothelial growth factor. ^a^ higher expression in lymphatic EC as compared to normal EC. ^b^ Cells cultured in 20% serum. ^c^ Differential effects on specific splice variants.

**Table 2:** Effect of galectins on endothelial cell function and angiogenesis *.

Process	Galectin-1	Galectin-3	Galectin-8	Galectin-9M ^f^
Activation	↑	↑	↑ ^a^	UNK
Proliferation	↑/↓^b^	↑	=	↓/= ^d^
Migration	↑/↓^b^	↑	↑	↑/↓ ^b,d^
Tube formation	↑	↑	↑/↓ ^c^	↑/= ^d^
Sprouting	↑	↑	↑	=
Permeability	↑	UNK	↑	UNK
Angiogenesis in vivo	↑	↑	↑	↓ ^e^

* Some effects are based on single studies and require additional confirmation. In addition, as described in the text, effects might be dependent on specific experimental conditions, galectin isoforms, or on the endothelial cell phenotype. UNK, unknown; ^a^ inflammatory activation; ^b^ concentration dependent, i.e., stimulation in low nM range and inhibition in high nM/low μM range. ^c^ Dependent on lymph (↓) vs. regular (↑) endothelial cell phenotype; ^d^ dependent on cell activation status; ^e^ only at high dose (500 nM). ^f^ Different effects were found for the separate domains of galectin-9 (see [23]).

## Data Availability

Not applicable.

## References

[1] Galley H.F., Webster N.R. (2004). Physiology of the endothelium. Br. J. Anaesth..

[2] Potente M., Gerhardt H., Carmeliet P. (2011). Basic and therapeutic aspects of angiogenesis. Cell.

[3] Folkman J. (1971). Tumor angiogenesis: Therapeutic implications. N. Engl. J. Med..

[4] Folkman J., Merler E., Abernathy C., Williams G. (1971). Isolation of a tumor factor responsible for angiogenesis. J. Exp. Med..

[5] Folkman J. (1972). Anti-Angiogenesis: New concept for therapy of solid tumors. Ann. Surg..

[6] Hanahan D., Weinberg R.A. (2011). Hallmarks of cancer: The next generation. Cell.

[7] Carmeliet P., Jain R.K. (2011). Molecular mechanisms and clinical applications of angiogenesis. Nature.

[8] Griffioen A.W., Thijssen V.L. (2014). Galectins in tumor angiogenesis. Ann. Transl. Med..

[9] Thijssen V.L., Rabinovich G.A., Griffioen A.W. (2013). Vascular galectins: Regulators of tumor progression and targets for cancer therapy. Cytokine Growth Factor Rev..

[10] Elola M.T., Ferragut F., Méndez-Huergo S.P., Croci D.O., Bracalente C., Rabinovich G.A. (2018). Galectins: Multitask signaling molecules linking fibroblast, endothelial and immune cell programs in the tumor microenvironment. Cell Immunol..

[11] Johannes L., Jacob R., Leffler H. (2018). Galectins at a glance. J. Cell Sci..

[12] Vasta G.R. (2012). Galectins as pattern recognition receptors: Structure, function, and evolution. Adv. Exp. Med. Biol..

[13] Hirabayashi J., Hashidate T., Arata Y., Nishi N., Nakamura T., Hirashima M., Urashima T., Oka T., Futai M., Muller W.E. (2002). Oligosaccharide specificity of galectins: A search by frontal affinity chromatography. Biochim. Biophys. Acta.

[14] Thijssen V.L., Hulsmans S., Griffioen A.W. (2008). The galectin profile of the endothelium: Altered expression and localization in activated and tumor endothelial cells. Am. J. Pathol..

[15] Lotan R., Belloni P.N., Tressler R.J., Lotan D., Xu X.C., Nicolson G.L. (1994). Expression of galectins on microvessel endothelial cells and their involvement in tumour cell adhesion. Glycoconj. J..

[16] Baum L.G., Seilhamer J.J., Pang M., Levine W.B., Beynon D., Berliner J.A. (1995). Synthesis of an endogeneous lectin, galectin-1, by human endothelial cells is up-regulated by endothelial cell activation. Glycoconj. J..

[17] Clausse N., van den Brule F., Waltregny D., Garnier F., Castronovo V. (1999). Galectin-1 expression in prostate tumor-associated capillary endothelial cells is increased by prostate carcinoma cells and modulates heterotypic cell-cell adhesion. Angiogenesis.

[18] Shekhar M.P., Nangia-Makker P., Tait L., Miller F., Raz A. (2004). Alterations in galectin-3 expression and distribution correlate with breast cancer progression: Functional analysis of galectin-3 in breast epithelial-endothelial interactions. Am. J. Pathol..

[19] Cueni L.N., Detmar M. (2009). Galectin-8 interacts with podoplanin and modulates lymphatic endothelial cell functions. Exp. Cell Res..

[20] Cardenas Delgado V.M., Nugnes L.G., Colombo L.L., Troncoso M.F., Fernandez M.M., Malchiodi E.L., Frahm I., Croci D.O., Compagno D., Rabinovich G.A. (2011). Modulation of endothelial cell migration and angiogenesis: A novel function for the “tandem-repeat” lectin galectin-8. FASEB J..

[21] Cattaneo V., Tribulatti M.V., Carabelli J., Carestia A., Schattner M., Campetella O. (2014). Galectin-8 elicits pro-inflammatory activities in the endothelium. Glycobiology.

[22] Heusschen R., Schulkens I.A., van Beijnum J., Griffioen A.W., Thijssen V.L. (2014). Endothelial LGALS9 splice variant expression in endothelial cell biology and angiogenesis. Biochim. Biophys. Acta.

[23] Aanhane E., Schulkens I.A., Heusschen R., Castricum K., Leffler H., Griffioen A.W., Thijssen V.L. (2018). Different angioregulatory activity of monovalent galectin-9 isoforms. Angiogenesis.

[24] Chakraborty A., Staudinger C., King S.L., Erickson F.C., Lau L.S., Bernasconi A., Luscinskas F.W., Perlyn C., Dimitroff C.J. (2021). Galectin-9 bridges human B cells to vascular endothelium while programming regulatory pathways. J. Autoimmun..

[25] Hepp P., Unverdorben L., Hutter S., Kuhn C., Ditsch N., Groß E., Mahner S., Jeschke U., Knabl J., Heidegger H.H. (2020). Placental Galectin-2 Expression in Gestational Diabetes: A Systematic, Histological Analysis. Int. J. Mol. Sci..

[26] Chen W.S., Cao Z., Sugaya S., Lopez M.J., Sendra V.G., Laver N., Leffler H., Nilsson U.J., Fu J., Song J. (2016). Pathological lymphangiogenesis is modulated by galectin-8-dependent crosstalk between podoplanin and integrin-associated VEGFR-3. Nat. Commun..

[27] Furuhata S., Ando K., Oki M., Aoki K., Ohnishi S., Aoyagi K., Sasaki H., Sakamoto H., Yoshida T., Ohnami S. (2007). Gene expression profiles of endothelial progenitor cells by oligonucleotide microarray analysis. Mol. Cell Biochem..

[28] Alam S., Li H., Margariti A., Martin D., Zampetaki A., Habi O., Cockerill G., Hu Y., Xu Q., Zeng L. (2011). Galectin-9 protein expression in endothelial cells is positively regulated by histone deacetylase 3. J. Biol. Chem..

[29] Imaizumi T., Kumagai M., Sasaki N., Kurotaki H., Mori F., Seki M., Nishi N., Fujimoto K., Tanji K., Shibata T. (2002). Interferon-{gamma} stimulates the expression of galectin-9 in cultured human endothelial cells. J. Leukoc. Biol..

[30] He J., Baum L.G. (2006). Endothelial cell expression of galectin-1 induced by prostate cancer cells inhibits T-cell transendothelial migration. Lab. Invest..

[31] Pranjol M.Z.I., Zinovkin D.A., Maskell A.R.T., Stephens L.J., Achinovich S.L., Los’ D.M., Nadyrov E.A., Hannemann M., Gutowski N.J., Whatmore J.L. (2019). Cathepsin L-induced galectin-1 may act as a proangiogenic factor in the metastasis of high-grade serous carcinoma. J. Transl. Med..

[32] Ahrens I., Domeij H., Topcic D., Haviv I., Merivirta R.M., Agrotis A., Leitner E., Jowett J.B., Bode C., Lappas M. (2011). Successful in vitro expansion and differentiation of cord blood derived CD34+ cells into early endothelial progenitor cells reveals highly differential gene expression. PLoS ONE.

[33] Deo P., Glenn J.V., Powell L.A., Stitt A.W., Ames J.M. (2009). Upregulation of oxidative stress markers in human microvascular endothelial cells by complexes of serum albumin and digestion products of glycated casein. J. Biochem. Mol. Toxicol..

[34] Gil C.D., La M., Perretti M., Oliani S.M. (2006). Interaction of human neutrophils with endothelial cells regulates the expression of endogenous proteins annexin 1, galectin-1 and galectin-3. Cell Biol. Int..

[35] Thijssen V.L., Heusschen R., Caers J., Griffioen A.W. (2015). Galectin expression in cancer diagnosis and prognosis: A systematic review. Biochim. Biophys. Acta.

[36] Croci D.O., Cerliani J.P., Dalotto-Moreno T., Méndez-Huergo S.P., Mascanfroni I.D., Dergan-Dylon S., Toscano M.A., Caramelo J.J., García-Vallejo J.J., Ouyang J. (2014). Glycosylation-Dependent Lectin-Receptor Interactions Preserve Angiogenesis in Anti-VEGF Refractory Tumors. Cell.

[37] Croci D.O., Cerliani J.P., Pinto N.A., Morosi L.G., Rabinovich G.A. (2014). Regulatory role of glycans in the control of hypoxia-driven angiogenesis and sensitivity to anti-angiogenic treatment. Glycobiology.

[38] Maller S.M., Cagnoni A.J., Bannoud N., Sigaut L., Pérez Sáez J.M., Pietrasanta L.I., Yang R.Y., Liu F.T., Croci D.O., Di Lella S. (2020). An adipose tissue galectin controls endothelial cell function via preferential recognition of 3-fucosylated glycans. FASEB J..

[39] Chen C., Duckworth C.A., Fu B., Mark Pritchard D., Rhodes J.M., Yu L.-G. (2014). Circulating galectins -2, -4 and -8 in cancer patients make important contributions to the increased circulation of several cytokines and chemokines that promote angiogenesis and metastasis. Br. J. Cancer.

[40] Chen C., Duckworth C.A., Zhao Q., Pritchard D.M., Rhodes J.M., Yu L.-G. (2013). Increased circulation of galectin-3 in cancer induces secretion of metastasis-promoting cytokines from blood vascular endothelium. Clin. Cancer Res..

[41] Colomb F., Wang W., Simpson D., Zafar M., Beynon R., Rhodes J.M., Yu L.G. (2017). Galectin-3 interacts with the cell-surface glycoprotein CD146 (MCAM, MUC18) and induces secretion of metastasis-promoting cytokines from vascular endothelial cells. J. Biol. Chem..

[42] Spitzenberger F., Graessler J., Schroeder H.E. (2001). Molecular and functional characterization of galectin 9 mRNA isoforms in porcine and human cells and tissues. Biochimie.

[43] Friedel M., André S., Goldschmidt H., Gabius H.J., Schwartz-Albiez R. (2016). Galectin-8 enhances adhesion of multiple myeloma cells to vascular endothelium and is an adverse prognostic factor. Glycobiology.

[44] Balan V., Nangia-Makker P., Jung Y.S., Wang Y., Raz A. (2010). Galectin-3: A novel substrate for c-Abl kinase. Biochim. Biophys. Acta.

[45] Gao X., Liu J., Liu X., Li L., Zheng J. (2017). Cleavage and phosphorylation: Important post-translational modifications of galectin-3. Cancer Metastasis Rev..

[46] Davis C.M., Hiremath G., Wiktorowicz J.E., Soman K.V., Straub C., Nance C., Quintanilla N., Pazdrak K., Thakkar K., Olive A.P. (2016). Proteomic Analysis in Esophageal Eosinophilia Reveals Differential Galectin-3 Expression and S-Nitrosylation. Digestion.

[47] Berbís M.Á., André S., Cañada F.J., Pipkorn R., Ippel H., Mayo K.H., Kübler D., Gabius H.J., Jiménez-Barbero J. (2014). Peptides derived from human galectin-3 N-terminal tail interact with its carbohydrate recognition domain in a phosphorylation-dependent manner. Biochem. Biophys. Res. Commun..

[48] Balan V., Nangia-Makker P., Kho D.H., Wang Y., Raz A. (2012). Tyrosine-phosphorylated galectin-3 protein is resistant to prostate-specific antigen (PSA) cleavage. J. Biol. Chem..

[49] Prudova A., auf dem Keller U., Butler G.S., Overall C.M. (2010). Multiplex N-terminome analysis of MMP-2 and MMP-9 substrate degradomes by iTRAQ-TAILS quantitative proteomics. Mol. Cell. Proteomics..

[50] Mazurek N., Conklin J., Byrd J.C., Raz A., Bresalier R.S. (2000). Phosphorylation of the beta-galactoside-binding protein galectin-3 modulates binding to its ligands. J. Biol. Chem..

[51] Nishi N., Itoh A., Shoji H., Miyanaka H., Nakamura T. (2006). Galectin-8 and galectin-9 are novel substrates for thrombin. Glycobiology.

[52] Nangia-Makker P., Wang Y., Raz T., Tait L., Balan V., Hogan V., Raz A. (2010). Cleavage of galectin-3 by matrix metalloproteases induces angiogenesis in breast cancer. Int. J. Cancer.

[53] Gabius H.J., Brehler R., Schauer A., Cramer F. (1986). Localization of endogenous lectins in normal human breast, benign breast lesions and mammary carcinomas. Virchows Arch. B Cell Pathol. Incl. Mol. Pathol..

[54] Patterson R.J., Wang W., Wang J.L. (2004). Understanding the biochemical activities of galectin-1 and galectin-3 in the nucleus. Glycoconj. J..

[55] Juszczynski P., Ouyang J., Monti S., Rodig S.J., Takeyama K., Abramson J., Chen W., Kutok J.L., Rabinovich G.A., Shipp M.A. (2007). The AP1-dependent secretion of galectin-1 by Reed Sternberg cells fosters immune privilege in classical Hodgkin lymphoma. Proc. Natl. Acad. Sci. USA.

[56] Schattner M., Rabinovich G.A. (2013). Galectins: New agonists of platelet activation. Biol. Chem..

[57] Troncoso M.F., Ferragut F., Bacigalupo M.L., Cárdenas Delgado V.M., Nugnes L.G., Gentilini L., Laderach D., Wolfenstein-Todel C., Compagno D., Rabinovich G.A. (2014). Galectin-8: A matricellular lectin with key roles in angiogenesis. Glycobiology.

[58] Funasaka T., Raz A., Nangia-Makker P. (2014). Galectin-3 in angiogenesis and metastasis. Glycobiology.

[59] Thijssen V.L., Griffioen A.W. (2014). Galectin-1 and galectin-9 in angiogenesis; A sweet couple. Glycobiology.

[60] Nambiar D.K., Aguilera T., Cao H., Kwok S., Kong C., Bloomstein J., Wang Z., Rangan V.S., Jiang D., von Eyben R. (2019). Galectin-1-driven T cell exclusion in the tumor endothelium promotes immunotherapy resistance. J. Clin. Invest..

[61] Whitney P., Maxwell S., Ryan U., Massaro D. (1985). Synthesis and binding of lactose-specific lectin by isolated lung cells. Am. J. Physiol..

[62] Allen H.J., Sucato D., Gottstine S., Kisailus E., Nava H., Petrelli N., Castillo N., Wilson D. (1991). Localization of endogenous beta-galactoside-binding lectin in human cells and tissues. Tumour Biol..

[63] Thijssen V.L., Postel R., Brandwijk R.J., Dings R.P., Nesmelova I., Satijn S., Verhofstad N., Nakabeppu Y., Baum L.G., Bakkers J. (2006). Galectin-1 is essential in tumor angiogenesis and is a target for antiangiogenesis therapy. Proc. Natl. Acad. Sci. USA.

[64] Hsieh S.H., Ying N.W., Wu M.H., Chiang W.F., Hsu C.L., Wong T.Y., Jin Y.T., Hong T.M., Chen Y.L. (2008). Galectin-1, a novel ligand of neuropilin-1, activates VEGFR-2 signaling and modulates the migration of vascular endothelial cells. Oncogene.

[65] Thijssen V.L., Barkan B., Shoji H., Aries I.M., Mathieu V., Deltour L., Hackeng T.M., Kiss R., Kloog Y., Poirier F. (2010). Tumor cells secrete galectin-1 to enhance endothelial cell activity. Cancer Res..

[66] van Beijnum J.R., Thijssen V.L., Läppchen T., Wong T.J., Verel I., Engbersen M., Schulkens I.A., Rossin R., Grüll H., Griffioen A.W. (2016). A key role for galectin-1 in sprouting angiogenesis revealed by novel rationally designed antibodies. Int. J. Cancer.

[67] Laderach D.J., Gentilini L.D., Giribaldi L., Delgado V.C., Nugnes L., Croci D.O., Al Nakouzi N., Sacca P., Casas G., Mazza O. (2013). A Unique Galectin Signature in Human Prostate Cancer Progression Suggests Galectin-1 as a Key Target for Treatment of Advanced Disease. Cancer Res..

[68] Croci D.O., Salatino M., Rubinstein N., Cerliani J.P., Cavallin L.E., Leung H.J., Ouyang J., Ilarregui J.M., Toscano M.A., Domaica C.I. (2012). Disrupting galectin-1 interactions with N-glycans suppresses hypoxia-driven angiogenesis and tumorigenesis in Kaposi’s sarcoma. J. Exp. Med..

[69] Tang D., Gao J., Wang S., Ye N., Chong Y., Huang Y., Wang J., Li B., Yin W., Wang D. (2015). Cancer-associated fibroblasts promote angiogenesis in gastric cancer through galectin-1 expression. Tumour Biol..

[70] Pérez Sáez J.M., Hockl P.F., Cagnoni A.J., Méndez Huergo S.P., García P.A., Gatto S.G., Cerliani J.P., Croci D.O., Rabinovich G.A. (2021). Characterization of a neutralizing anti-human galectin-1 monoclonal antibody with angioregulatory and immunomodulatory activities. Angiogenesis.

[71] Cheng Y.H., Jiang Y.F., Qin C., Shang K., Yuan Y., Wei X.J., Xu Z., Luo X., Wang W., Qu W.S. (2021). Galectin-1 Contributes to Vascular Remodeling and Blood Flow Recovery After Cerebral Ischemia in Mice. Transl. Stroke Res..

[72] D’Haene N., Sauvage S., Maris C., Adanja I., Le Mercier M., Decaestecker C., Baum L., Salmon I. (2013). VEGFR1 and VEGFR2 Involvement in Extracellular Galectin-1- and Galectin-3-Induced Angiogenesis. PLoS ONE.

[73] Wei J., Li D.K., Hu X., Cheng C., Zhang Y. (2021). Galectin-1-RNA interaction map reveals potential regulatory roles in angiogenesis. FEBS Lett..

[74] Park J.W., Voss P.G., Grabski S., Wang J.L., Patterson R.J. (2001). Association of galectin-1 and galectin-3 with Gemin4 in complexes containing the SMN protein. Nucleic Acids Res..

[75] Wu M.H., Ying N.W., Hong T.M., Chiang W.F., Lin Y.T., Chen Y.L. (2014). Galectin-1 induces vascular permeability through the neuropilin-1/vascular endothelial growth factor receptor-1 complex. Angiogenesis.

[76] Jouve N., Despoix N., Espeli M., Gauthier L., Cypowyj S., Fallague K., Schiff C., Dignat-George F., Vély F., Leroyer A.S. (2013). The involvement of CD146 and its novel ligand Galectin-1 in apoptotic regulation of endothelial cells. J. Biol. Chem..

[77] Leffler H., Carlsson S., Hedlund M., Qian Y., Poirier F. (2004). Introduction to galectins. Glycoconj. J..

[78] Perillo N.L., Pace K.E., Seilhamer J.J., Baum L.G. (1995). Apoptosis of T cells mediated by galectin-1. Nature.

[79] Cho M., Cummings R.D. (1996). Characterization of monomeric forms of galectin-1 generated by site-directed mutagenesis. Biochemistry.

[80] Salomonsson E., Larumbe A., Tejler J., Tullberg E., Rydberg H., Sundin A., Khabut A., Frejd T., Lobsanov Y.D., Rini J.M. (2010). Monovalent interactions of galectin-1. Biochemistry.

[81] Adams L., Scott G.K., Weinberg C.S. (1996). Biphasic modulation of cell growth by recombinant human galectin-1. Biochim. Biophys. Acta.

[82] Vas V., Fajka-Boja R., Ion G., Dudics V., Monostori E., Uher F. (2005). Biphasic effect of recombinant galectin-1 on the growth and death of early hematopoietic cells. Stem Cells.

[83] Camby I., Le Mercier M., Lefranc F., Kiss R. (2006). Galectin-1: A small protein with major functions. Glycobiology.

[84] Nangia-Makker P., Honjo Y., Sarvis R., Akahani S., Hogan V., Pienta K.J., Raz A. (2000). Galectin-3 induces endothelial cell morphogenesis and angiogenesis. Am. J. Pathol..

[85] Nangia-Makker P., Hogan V., Honjo Y., Baccarini S., Tait L., Bresalier R., Raz A. (2002). Inhibition of human cancer cell growth and metastasis in nude mice by oral intake of modified citrus pectin. J. Natl. Cancer Inst..

[86] Markowska A.I., Liu F.T., Panjwani N. (2010). Galectin-3 is an important mediator of VEGF- and bFGF-mediated angiogenic response. J. Exp. Med..

[87] Markowska A.I., Jefferies K.C., Panjwani N. (2011). Galectin-3 protein modulates cell surface expression and activation of vascular endothelial growth factor receptor 2 in human endothelial cells. J. Biol. Chem..

[88] Wesley U.V., Vemuganti R., Ayvaci E.R., Dempsey R.J. (2013). Galectin-3 enhances angiogenic and migratory potential of microglial cells via modulation of integrin linked kinase signaling. Brain Res..

[89] Wan S.Y., Zhang T.F., Ding Y. (2011). Galectin-3 enhances proliferation and angiogenesis of endothelial cells differentiated from bone marrow mesenchymal stem cells. Transplant. Proc..

[90] Ou H.C., Chou W.C., Hung C.H., Chu P.M., Hsieh P.L., Chan S.H., Tsai K.L. (2019). Galectin-3 aggravates ox-LDL-induced endothelial dysfunction through LOX-1 mediated signaling pathway. Environ. Toxicol..

[91] Zhang L., Li Y.M., Zeng X.X., Wang X.Y., Chen S.K., Gui L.X., Lin M.J. (2018). Galectin-3- Mediated Transdifferentiation of Pulmonary Artery Endothelial Cells Contributes to Hypoxic Pulmonary Vascular Remodeling. Cell Physiol. Biochem..

[92] Dos Santos S.N., Sheldon H., Pereira J.X., Paluch C., Bridges E.M., El-Cheikh M.C., Harris A.L., Bernardes E.S. (2017). Galectin-3 acts as an angiogenic switch to induce tumor angiogenesis via Jagged-1/Notch activation. Oncotarget.

[93] Jia W., Wang Z., Gao C., Wu J., Wu Q. (2021). Trajectory modeling of endothelial-to-mesenchymal transition reveals galectin-3 as a mediator in pulmonary fibrosis. Cell Death Dis..

[94] Mirandola L., Yu Y., Chui K., Jenkins M.R., Cobos E., John C.M., Chiriva-Internati M. (2011). Galectin-3C inhibits tumor growth and increases the anticancer activity of bortezomib in a murine model of human multiple myeloma. PLoS ONE.

[95] Yang E., Shim J.S., Woo H.J., Kim K.W., Kwon H.J. (2007). Aminopeptidase N/CD13 induces angiogenesis through interaction with a pro-angiogenic protein, galectin-3. Biochem. Biophys. Res. Commun..

[96] Fukushi J., Makagiansar I.T., Stallcup W.B. (2004). NG2 proteoglycan promotes endothelial cell motility and angiogenesis via engagement of galectin-3 and alpha3beta1 integrin. Mol. Biol. Cell.

[97] Sedlář A., Trávníčková M., Bojarová P., Vlachová M., Slámová K., Křen V., Bačáková L. (2021). Interaction between Galectin-3 and Integrins Mediates Cell-Matrix Adhesion in Endothelial Cells and Mesenchymal Stem Cells. Int. J. Mol. Sci..

[98] Gallardo-Vara E., Ruiz-Llorente L., Casado-Vela J., Ruiz-Rodríguez M.J., López-Andrés N., Pattnaik A.K., Quintanilla M., Bernabeu C. (2019). Endoglin Protein Interactome Profiling Identifies TRIM21 and Galectin-3 as New Binding Partners. Cells.

[99] Zhang Z., Zheng Y., Wang H., Zhou Y., Tai G. (2018). CD146 interacts with galectin-3 to mediate endothelial cell migration. FEBS Lett..

[100] Hadari Y.R., Arbel-Goren R., Levy Y., Amsterdam A., Alon R., Zakut R., Zick Y. (2000). Galectin-8 binding to integrins inhibits cell adhesion and induces apoptosis. J. Cell Sci..

[101] Zamorano P., Koning T., Oyanadel C., Mardones G.A., Ehrenfeld P., Boric M.P., González A., Soza A., Sánchez F.A. (2019). Galectin-8 induces endothelial hyperpermeability through the eNOS pathway involving S-nitrosylation-mediated adherens junction disassembly. Carcinogenesis.

[102] Eshkar Sebban L., Ronen D., Levartovsky D., Elkayam O., Caspi D., Aamar S., Amital H., Rubinow A., Golan I., Naor D. (2007). The involvement of CD44 and its novel ligand galectin-8 in apoptotic regulation of autoimmune inflammation. J. Immunol..

[103] Forster-Horvath C., Meszaros L., Raso E., Dome B., Ladanyi A., Morini M., Albini A., Timar J. (2004). Expression of CD44v3 protein in human endothelial cells in vitro and in tumoral microvessels in vivo. Microvasc. Res..

[104] Griffioen A.W., Coenen M.J., Damen C.A., Hellwig S.M., van Weering D.H., Vooys W., Blijham G.H., Groenewegen G. (1997). CD44 is involved in tumor angiogenesis; an activation antigen on human endothelial cells. Blood.

[105] Martín-Villar E., Fernández-Muñoz B., Parsons M., Yurrita M.M., Megías D., Pérez-Gómez E., Jones G.E., Quintanilla M. (2010). Podoplanin associates with CD44 to promote directional cell migration. Mol. Biol. Cell.

[106] Varinská L., Fáber L., Petrovová E., Balážová L., Ivančová E., Kolář M., Gál P. (2020). Galectin-8 Favors VEGF-Induced Angiogenesis: In Vitro Study in Human Umbilical Vein Endothelial Cells and In Vivo Study in Chick Chorioallantoic Membrane. Anticancer. Res..

[107] Matsumoto R., Matsumoto H., Seki M., Hata M., Asano Y., Kanegasaki S., Stevens R.L., Hirashima M. (1998). Human ecalectin, a variant of human galectin-9, is a novel eosinophil chemoattractant produced by T lymphocytes. J. Biol. Chem..

[108] Imaizumi T., Yoshida H., Nishi N., Sashinami H., Nakamura T., Hirashima M., Ohyama C., Itoh K., Satoh K. (2007). Double-stranded RNA induces galectin-9 in vascular endothelial cells: Involvement of TLR3, PI3K, and IRF3 pathway. Glycobiology.

[109] Ishikawa A., Imaizumi T., Yoshida H., Nishi N., Nakamura T., Hirashima M., Satoh K. (2004). Double-stranded RNA enhances the expression of galectin-9 in vascular endothelial cells. Immunol Cell Biol..

[110] Hirashima M., Kashio Y., Nishi N., Yamauchi A., Imaizumi T.A., Kageshita T., Saita N., Nakamura T. (2004). Galectin-9 in physiological and pathological conditions. Glycoconj. J..

[111] Heusschen R., Griffioen A.W., Thijssen V.L. (2013). Galectin-9 in tumor biology: A jack of multiple trades. Biochim. Biophys. Acta.

[112] O’Brien M.J., Shu Q., Stinson W.A., Tsou P.S., Ruth J.H., Isozaki T., Campbell P.L., Ohara R.A., Koch A.E., Fox D.A. (2018). A unique role for galectin-9 in angiogenesis and inflammatory arthritis. Arthritis Res. Ther..

[113] Miller M.C., Ludwig A.K., Wichapong K., Kaltner H., Kopitz J., Gabius H.J., Mayo K.H. (2018). Adhesion/growth-regulatory galectins tested in combination: Evidence for formation of hybrids as heterodimers. Biochem. J..

[114] Drobnjak T., Jónsdóttir A.M., Helgadóttir H., Runólfsdóttir M.S., Meiri H., Sammar M., Osol G., Mandalà M., Huppertz B., Gizurarson S. (2019). Placental protein 13 (PP13) stimulates rat uterine vessels after slow subcutaneous administration. Int. J. Womens Health.

[115] Etulain J., Negrotto S., Tribulatti M.V., Croci D.O., Carabelli J., Campetella O., Rabinovich G.A., Schattner M. (2014). Control of angiogenesis by galectins involves the release of platelet-derived proangiogenic factors. PLoS ONE.

[116] Eckardt V., Miller M.C., Blanchet X., Duan R., Leberzammer J., Duchene J., Soehnlein O., Megens R.T., Ludwig A.K., Dregni A. (2020). Chemokines and galectins form heterodimers to modulate inflammation. EMBO Rep..

[117] Gordon-Alonso M., Hirsch T., Wildmann C., van der Bruggen P. (2017). Galectin-3 captures interferon-gamma in the tumor matrix reducing chemokine gradient production and T-cell tumor infiltration. Nat. Commun..

[118] Nagarsheth N., Wicha M.S., Zou W. (2017). Chemokines in the cancer microenvironment and their relevance in cancer immunotherapy. Nat. Rev. Immunol..

[119] Zlotnik A., Yoshie O. (2012). The chemokine superfamily revisited. Immunity.

[120] Rabinovich G.A., Croci D.O. (2012). Regulatory circuits mediated by lectin-glycan interactions in autoimmunity and cancer. Immunity.

[121] Schulkens I.A., Griffioen A.W., Thijssen V.L., Klyosov A. (2012). ACS Symposium Series: Galectins and Disease Implications for Targeted Therapeutics.

[122] Rabinovich G.A., Cumashi A., Bianco G.A., Ciavardelli D., Iurisci I., D’Egidio M., Piccolo E., Tinari N., Nifantiev N., Iacobelli S. (2006). Synthetic lactulose amines: Novel class of anticancer agents that induce tumor-cell apoptosis and inhibit galectin-mediated homotypic cell aggregation and endothelial cell morphogenesis. Glycobiology.

[123] Ito K., Scott S.A., Cutler S., Dong L.F., Neuzil J., Blanchard H., Ralph S.J. (2011). Thiodigalactoside inhibits murine cancers by concurrently blocking effects of galectin-1 on immune dysregulation, angiogenesis and protection against oxidative stress. Angiogenesis.

[124] Dings R.P.M., Miller M.C., Nesmelova I., Astorgues-Xerri L., Kumar N., Serova M., Chen X., Raymond E., Hoye T.R., Mayo K.H. (2012). Antitumor agent calixarene 0118 targets human galectin-1 as an allosteric inhibitor of carbohydrate binding. J. Med. Chem..

